# Medial condyle hypoplasia in adolescent and young adult patients with trochlear dysplasia: a retrospective study

**DOI:** 10.1590/0100-3984.2023.0051

**Published:** 2023

**Authors:** Sruthi Jacob, Harshavardhan Mahalingam

**Affiliations:** 1 Department of Radiodiagnosis, Sri Ramachandra Institute of Higher Education and Research, Chennai, Tamil Nadu, India.

**Keywords:** Joint instability, Patella, Femur, Knee joint, Patellar dislocation, Magnetic resonance imaging, Instabilidade articular, Patela; Fêmur, Articulação do joelho, Luxação patelar, Ressonância magnética

## Abstract

**Objective:**

To determine the association between medial femoral condyle hypoplasia and trochlear dysplasia by analyzing the knee magnetic resonance imaging scans of young patients with or without trochlear dysplasia.

**Materials and Methods:**

This was a retrospective analysis of magnetic resonance imaging scans of the knees of young individuals (16-35 years of age): 30 patients with trochlear dysplasia and 30 individuals with no signs of patellofemoral instability. The ratios between the depth, width, and height of the medial and lateral femoral condyles (dLC/dMC, wLC/wMC, and hLC/hMC, respectively) were calculated, as was the ratio between the width of the medial condyle and the total width of the femur (wMC/FW). All of the values were determined in consensus by two radiologists.

**Results:**

We evaluated 60 patients: 30 with trochlear dysplasia and 30 without. The mean dLC/dMC, wLC/wMC, and hLC/hMC ratios were higher in the patients than in the controls (*p* < 0.05), whereas the mean wMC/FW ratio was lower in the patients (*p* < 0.05). The optimal cutoff values, obtained by calculating the areas under the receiver operating characteristic curves, were 1.0465 for the dLC/dMC ratio (76% sensitivity and 63.3% specificity), 0.958 for the wLC/wMC ratio (80% sensitivity and 73.3% specificity), and 1.080 for the hLC/hMC ratio (93.3% sensitivity and 93.3% specificity).

**Conclusion:**

Our findings confirm our hypothesis that trochlear dysplasia is associated with medial condyle hypoplasia.

## Abstract (pt)

**Objetivo:**

Determinar a associação entre hipoplasia do côndilo femoral medial e displasia troclear mediante análise de ressonância magnética do joelho de pacientes jovens com displasia troclear e sem displasia troclear.

**Materiais e Métodos:**

Análise retrospectiva de exames de ressonância magnética de joelhos de indivíduos jovens (16 a 35 anos de idade), sendo 30 pacientes com displasia troclear e 30 indivíduos sem sinais de instabilidade femoropatelar. As razões entre a profundidade, largura e altura dos côndilos femorais mediais e laterais (dLC/dMC, wLC/wMC e hLC/hMC, respectivamente) foram calculadas, assim como a razão entre a largura do côndilo medial e a largura total do fêmur (wMC/FW). Todos os valores foram determinados em consenso por dois radiologistas

**Resultados:**

Foram incluídos no estudo 60 adolescentes e adultos jovens, 30 com displasia troclear e 30 sem displasia troclear. A média das razões dLC/dMC, wLC/wMC, hLC/hMC foi maior nos pacientes do que nos controles (*p* < 0,05), enquanto a média da razão wMC/FW foi menor nos pacientes (*p* < 0,05). Os valores de corte ótimos obtidos da área sob a curva característica de operação do receptor foram 1,0465 para dLC/dMC (sensibilidade de 76% e especificidade de 63,3%), 0,958 para wLC/wMC (sensibilidade de 80% e especificidade de 73,3%) e 1,080 para hLC/hMC (sensibilidade de 93,3% e especificidade de 93,3%).

**Conclusão:**

Nossos resultados confirmam nossa hipótese de que a displasia troclear está associada a hipoplasia do côndilo medial.

## INTRODUCTION

Patellofemoral instability or patellar maltracking, produced by incongruence between the patella and the trochlear groove, results in a tendency for recurrent patellar dislocation. Patellofemoral instability ranges from mild maltracking to obvious lateral patellar dislocation^(1)^. Patellar instability typically affects young active individuals. In such individuals, the usual presentation is anterior knee pain, traumatic lateral patellar dislocation, or recurrent patellar instability. The patellofemoral joint is stabilized by two types of stabilizers: active and passive. The active stabilizers are extensor muscles, and the passive stabilizers are bones and ligaments. Lateral patellar dislocation is caused by medial stabilizer injury, leading to the patella impacting the lateral femoral condyle, which results in the so-called kissing lesions^(1)^. The morphological abnormalities predisposing to patellar instability are trochlear dysplasia, patella alta, and lateralization of the tibial tuberosity. Patellar maltracking occurs with a frequency similar to that of meniscal lesions^(2)^.

Approximately 96% of patients presenting with patellofemoral instability are found to have trochlear dysplasia, which is considered one of the main risk factors for patellofemoral instability^(3,4)^. Magnetic resonance imaging (MRI) is the diagnostic tool of choice in cases of patellofemoral instability and for evaluating trochlear dysplasia^(3,5)^. Early evaluation of these patients is necessary because of the long term risk of progressive articular cartilage damage and advanced osteoarthritis^(1)^.

Various quantitative parameters have traditionally been used to confirm trochlear dysplasia, namely the lateral trochlear inclination angle, sulcus angle, trochlear facet asymmetry, and trochlear depth. A lateral trochlear inclination angle < 11° is an indicator of trochlear dysplasia, with 87% specificity and 93% sensitivity^(1,6)^. Another indicator of trochlear dysplasia is a trochlear facet ratio < 0.4, which has a specificity of 96% and a sensitivity of 100%^(1,6)^. Additional indicators are a sulcus angle > 145–150° and a trochlear depth < 3 mm. One finding described on lateral radiographs of the knee in cases of trochlear dysplasia is the crossing sign, in which the line representing the deepest portion of the trochlear groove crosses the anterior border of two condyles. That sign is a predictor of patellar dislocation^(7)^.

Although the diagnosis of trochlear dysplasia is well established, the etiology of trochlear dysplasia is still under debate. It has been proposed that it could be a genetic/congenital disorder, given that the cartilaginous trochlear sulcus is formed *in utero*^(8,9)^ It has also been proposed that the mechanical load on the knee joint during childhood has an effect on trochlear development^(10)^.

On visual inspection of the MRI scans of patients presenting with recurrent patellar dislocation, the medial condyle of the femur appears smaller than usual. In trochlear dysplasia, medial trochlear facet hypoplasia is a proven morphological parameter, whereas medial femoral condyle hypoplasia is a factor that has rarely been assessed. Below-normal height of the medial femoral condyle has also been described in trochlear dysplasia, as has a flat trochlea^(11)^. However, there have been few studies demonstrating medial femoral condyle hypoplasia in cases of trochlear dysplasia. We hypothesized that medial femoral condyle hypoplasia would be one of the factors associated with trochlear dysplasia.

## MATERIALS AND METHODS

This was a retrospective analysis of the MRI scans of 60 knees, 30 from patients with trochlear dysplasia and 30 from individuals with no radiological or clinical signs of patellofemoral instability, acquired between January of 2020 and March of 2023. The study group comprised 18 females and 12 males, whereas the control group comprised 12 females and 18 males. In the sample as a whole, ages ranged from 16 to 35 years.

The sample size required in each arm of the study was calculated according to the following formula:


$$sample\;size\left(N\right)=1+2{\left(Z_{\alpha }+Z_{1-\beta }\right)}^{2}\alpha^{2}/\delta^{2}$$

where Z_α_ is the standard normal distribution for a type I (a) error, Ζ_1−β_ is the standard normal distribution for 1 minus a type 2 (β) error, σ is the pooled standard deviation, and δ is the difference between the means. Thus, the sample size required was determined to be 30 subjects per group, assuming a power of 90% and a 95% confidence interval.

The patients in study group were included on the basis of clinical and radiological criteria. All had clinical features of patellar instability in the form of one of the following: a history of recurrent patellar dislocation or anterior knee pain; or an unstable feeling with a positive apprehension test result or an abnormal Q angle. On imaging, all of these patients had an abnormal sulcus angle (> 145°) or a shallow bony trochlear sulcus (< 5 mm). We included adolescents and young adults who had trochlear dysplasia, regardless of the stage of dysplasia.

The MRI knee examinations included in control group we selected from among those of individuals referred to our institute for the evaluation of other knee pathologies, with normal sulcus angles and depths, in whom the knee MRI findings were near normal. We excluded the scans of individuals with major ligament tears, meniscal injuries, fractures, tumors, or advanced degenerative changes.

**Figure 1:**
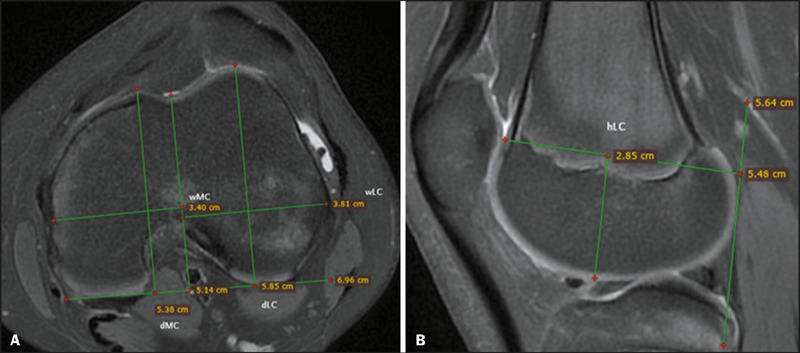
Measurements of the knee joint. Axial and sagittal fat-suppressed proton density-weighted MRI sequences **(A** and **B,** respectively).

Multiparametric images were assessed for calculating all of the parameters evaluated, including the depth of the lateral and medial femoral condyles (dLC and dMC, respectively); the width of the lateral and medial femoral condyles (wLC and wMC, respectively); the height of the lateral and medial femoral condyles (hLC and hMC, respectively); and femoral width (FW). For all measurements, axial and sagittal fat-suppressed proton density-weighted sequences, with 3-mm slices, were used. The axial sequence with the greatest anteroposterior and mediolateral extension was selected for measuring the dLC, dMC, wLC, and wMC, the posterior condylar line being taken as the reference line. The wLC and wMC were measured to the line along the deepest point of the trochlear groove. The total FW was also calculated in the same section. For measuring the hLC and hMC, the sagittal section of the fat-suppressed proton density-weighted sequence with the greatest anteroposterior extension at the condyle level was selected, the longitudinal posterior femoral condylar line being taken as the reference line. The hLC and hMC were drawn from the joint line to the dLC and dMC in the sagittal plane. All the measurements were made up to the cartilage level (Figure 1). All of the values were measured in consensus by two radiologists.

On the basis of the values obtained for the dLC, dMC, wLC, wMC, hLC, hMC, and FW, we calculated the dLC/dMC, wLC/wMC, and hLC/hMC ratios, which represent the anteroposterior dimension, mediolateral dimension, and craniocaudal dimension of the condyles, respectively, as well as the wMC/FW ratio, which represents the width of medial femoral condyle relative to the total width of the femur. All the measurements were compared between the two groups.

### Statistical analysis

Continuous data are presented as mean and standard deviation. The dLC/dMC, wLC/wMC, hLC/hMC, and wMC/FW ratios were compared between the groups by using paired t-tests. Statistical analyses were performed with the Statistical Package for the Social Sciences, version 16.0 (SPSS Inc., Chicago, IL, USA). Values of *p* < 0.05 were considered significant. Receiver operating characteristic (ROC) curves were generated for all four ratios, and the cutoff values with the best sensitivity and specificity were calculated, on the basis of the areas under the curve, for the dLC/dMC, wLC/wMC, and hLC/hMC ratios.

## RESULTS

**Figure 2:**
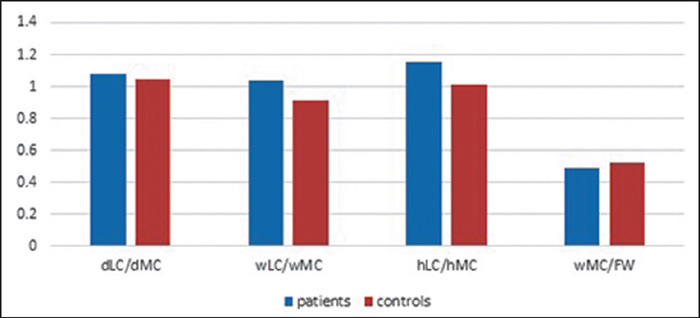
Bar graph comparing the dLC/dMC, wLC/wMC, hLC/hMC, and wMC/FW ratios between the patients with trochlear dysplasia and the control subjects.

The mean ages in the study group and control group were 24.5 ± 6.16 years and 23.6 ± 4.29 years, respectively. In the study group and control group, respectively, the mean dLC/dMC ratios were 1.07 ± 0.04 and 1.04 ± 0.03 (*p* = 0.0017); the mean wLC/wMC ratios were 1.03 ± 0.1 and 0.91 ± 0.08 (*p* < 0.00001); the mean hLC/hMC ratios were 1.15 ± 0.07 and 1.01 ± 0.06 (*p* < 0.00001); and the mean wMC/FW ratios were 0.49 ± 0.02 and 0.51 ± 0.01 (***p*** < 0.0001). For the ratios calculated, all of the intergroup differences were statistically significant (*p* < 0.05), as illustrated in Figure 2. The mean wMC/FW ratio was 0.49 ± 0.03 among the males and 0.49 ± 0.03 among the females **(*p*** = 0.966).

**Figure 3:**
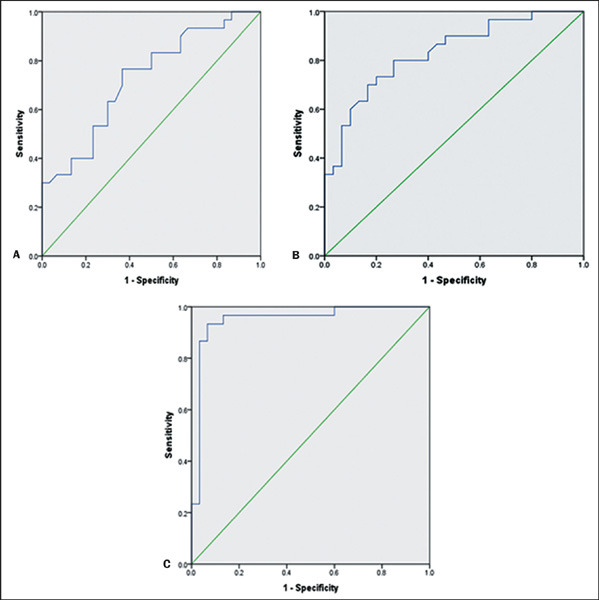
ROC curves for the dLC/dMC, wLC/wMC, and hLC/hMC ratios **(A, B,** and **C,** respectively).

The optimal cutoff values, in terms of sensitivity and specificity, were found to be 1.0465 for the dLC/dMC ratio (76% sensitivity and 63.3% specificity), 0.958 for the wLC/wMC ratio (80% sensitivity and 73.3% specificity), and 1.080 for the hLC/hMC ratio (93.3% sensitivity and 93.3% specificity), as depicted in Figure 3.

## DISCUSSION

Trochlear dysplasia is a morphological abnormality of the trochlear groove, characterized by a loss of normal concavity. Trochlear dysplasia, chondromalacia patella, and patellofemoral cartilage loss resulting in osteoarthritis all are interrelated^(12)^.

Two etiological factors are implicated in the development of trochlear dysplasia^(13)^: congenital genetic determination; and stress stimulation of the patella. Abnormal loading of the knee joint during childhood or adolescence, even loading that results from a surgical procedure, can also promote the development of trochlear dysplasia^(3)^. Breech presentation *in utero* is also considered to be a causative factor of trochlear dysplasia^(14)^.

To date, there have been few studies of bone and cartilage development in the trochlea. The sulcus angle is an important parameter for identifying trochlear dysplasia. The bony and cartilaginous sulcus angles can both be measured. The cartilaginous trochlear sulcus is almost fully developed (in adult form) at birth^(15,16)^. In an MRI study of normal pediatric knees, Trivellas et al.^(15)^ found that there was no significant variation in the cartilaginous sulcus angle with age, whereas the bony sulcus angle decreases with age, suggesting that underlying subchondral bone morphology alone changes with age. The authors also stated that the shape of the trochlea is primarily predetermined by genetics. In a study conducted by 0ye et al.^(17)^, ultrasound examination of the knee was performed at birth and repeated at 6, 18, and 72 months of age. They observed that a trochlea that is dysplastic at birth remains shallow until six years of age and that the sulcus angle shows a very small yet statistically significant decrease, while still remaining higher, suggesting trochlear dysplasia, whereas no significant morphological change was observed in newborns with normal knees. This suggests that the determinants of trochlear dysplasia are genetic rather than developmental. The higher sulcus angle seen in trochlear dysplasia (> 145°) is due to a shallow/flat trochlea and is also associated with patellar dislocation and osteochondral damage. A significant association has been observed between trochlear dysplasia and the incidence of osteochondral damage^(18)^.

The imaging studies used for the evaluation of patellar maltracking include radiographs, computed tomography, and MRI. The radiographs are taken mainly to look for osseous abnormalities like patella alta and trochlear dysplasia. To assess predisposing factors for patellar maltracking, such as trochlear dysplasia, patella alta, and lateralization of tibial tuberosity, MRI is the modality of choice^(1,19)^. Structural changes such as injury to the medial patellar retinaculum or medial patellofemoral ligament can also be assessed with MRI^(20)^.

Traditionally, the computed tomography- or MRI-based Dejour classification has been used for the grading of trochlear dysplasia, as follows^(21)^: type A (shallow trochlea and crossing sign in lateral view); type B (crossing sign, supratrochlear spur in lateral view, and convex or flat trochlea); type C (crossing sign, double contour sign, and hypoplastic medial condyle); or type D (crossing sign, double contour, supratrochlear spur, and a cliff-like pattern between trochlear facets). Another MRI-based classification system which was introduced recently is the Oswestry-Bristol classification^(22,23)^. Axial T2 weighted MRI images were used for grading—mild, moderate, and severe trochlear dysplasia, based on a shallow trochlea, flat trochlea, and convex trochlea.

In Dejour type C trochlear dysplasia, there is medial facet hypoplasia (facet asymmetry). On the basis of that classification, Stepanovich et al.^(24)^ found that a medial condyle trochlear facet < 1 mm is more likely to be associated with patellar instability. Although medial trochlear facet hypoplasia is a proven morphological feature in trochlear dysplasia^(6)^, medial femoral condyle hypoplasia as a whole or in relation to total femoral width is a poorly understood parameter. To date, there have been few studies of this topic^(1,3)^.

Kim et al.^(1)^ stated that higher grades of trochlear dysplasia are associated with medial femoral condyle hypoplasia, as well as with a flat or convex trochlea. The double contour sign on a lateral radiograph is due to a hypoplastic medial facet posterior to the lateral facet. The cliff pattern seen on axial images is due to asymmetry between the lateral and medial trochlear facets^(21)^.

The most important finding of our study was medial condyle hypoplasia among the cases of trochlear dysplasia. We observed that the dLC/dMC, wLC/wMC, and hLC/hMC ratios were higher in the study group than in the control group, suggesting that there was medial condyle hypoplasia in the study group patients. The wMC/FW ratio was lower in the study group, which is suggestive of the same morphological change. In a study of medial condyle hypoplasia in trochlear dysplasia, conducted by Keshmiri et al. ^(3)^, the sample included only cases of high-grade trochlear dysplasia (Dejour type B and above). In the present study, we included all cases of trochlear dysplasia, regardless of the severity, and found a significant association between trochlear dysplasia and medial condyle hypoplasia.

Biedert et al.^(25)^ stated that there are two major morphological types of trochlear dysplasia, one with decreased depth of the center/medial trochlear facet and another with decreased inclination of the lateral trochlear facet. They observed decreased lateral condyle height in five of the 30 cases evaluated, whereas that was not found in any of our study group patients. That discrepancy could be due to the fact that we employed three-dimensional height assessment, whereas those authors measured height in only one dimension.

Another important achievement of our study was the determination of the optimal cutoff values for the dLC/dMC, wLC/wMC, and hLC/hMC ratios. Because the hLC/hMC ratio cutoff was found to have the highest sensitivity and specificity, it should be the best parameter for differentiating between patients with trochlear dysplasia and those with normal trochlear morphology.

Comparing the results between the male and female patients with trochlear dysplasia, we found no significant difference in the wMC/FW ratio, which indicates the medial femoral condyle width relative to the total width. However, previous studies have shown that the medial and lateral femoral condyles are larger in males than in females^(25)^. In our sample, we observed no significant sex-related differences for any of the variables evaluated. That could be related to the relatively small size of our sample. The majority (60%) of our patients with trochlear dysplasia and patellofemoral instability were female. We observed that patellofemoral instability was more common among the females than among the males. That could be due to the fact that, morphologically, the trochleas of female knees are smaller than are those of male knees. However, in a recent study, that difference was not found to be significant after normalization for patient height^(26,27)^. Therefore, controversy still exists regarding why females are affected more by trochlear dysplasia; that is, whether it is due to the smaller condyle dimensions or to the wider, shallower trochlear groove. The smaller medial condyle in patients with patellar instability might have therapeutic implications, which could be addressed in future studies.

## CONCLUSION

Trochlear dysplasia is significantly associated with a smaller medial femoral condyle. The possibility that medial condyle hypoplasia is a contributing factor in the pathogenesis of trochlear dysplasia merits further study.
